# Association between gut microbiota and diabetic nephropathy: a mendelian randomization study

**DOI:** 10.3389/fmicb.2024.1309871

**Published:** 2024-03-27

**Authors:** Yongxiu Jin, Chenxi Han, Dongliang Yang, Shanlin Gao

**Affiliations:** ^1^Department of Nephrology, Tangshan Gongren Hosiptal, Tangshan, China; ^2^Graduate School, Hebei Medical University, Shijiazhuang, China; ^3^Tangshan Maternal and Child Health Hospital, Tangshan, China; ^4^Cangzhou Medical College, Cangzhou, China

**Keywords:** diabetic nephropathy, gut microbiota, mendelian randomization, short-chain fatty acids, insulin resistance

## Abstract

**Background:**

The correlation between diabetic nephropathy (DN) and gut microbiota (GM) has been suggested in numerous animal experiments and cross-sectional studies. However, a causal association between GM and DN has not been ascertained.

**Methods:**

This research adopted MR analysis to evaluate the causal link between GM and DN derived from data acquired through publicly available genome-wide association studies (GWAS). The study utilized the inverse variance weighted (IVW) approach to assess causal association between GM and DN. Four additional methods including MR-Egger, weighted median, weighted mode, and simple mode were employed to ensure comprehensive analysis and robust results. The Cochran’s Q test and the MR-Egger method were conducted to identify heterogeneity and horizontal pleiotropy, respectively. The leave-one-out approach was utilized to evaluate the stability of MR results. Finally, a reverse MR was performed to identify the reverse causal association between GM and DN.

**Results:**

According to IVW analysis, Class Verrucomicrobiae (*p* = 0.003), Order Verrucomicrobiales (*p* = 0.003), Family *Verrucomicrobiaceae* (*p* = 0.003), Genus *Akkermansia* (*p* = 0.003), Genus Catenibacterium (*p* = 0.031), Genus *Coprococcus* 1 (*p* = 0.022), Genus *Eubacterium hallii* group (*p* = 0.018), and Genus *Marvinbryantia* (*p* = 0.023) were associated with a higher risk of DN. On the contrary, Class *Actinobacteria* (*p* = 0.037), Group *Eubacterium ventriosum* group (*p* = 0.030), Group *Ruminococcus gauvreauii* group (*p* = 0.048), Order *Lactobacillales* (*p* = 0.045), Phylum *Proteobacteria* (*p* = 0.017) were associated with a lower risk of DN. The sensitivity analysis did not identify any substantial pleiotropy or heterogeneity in the outcomes. We found causal effects of DN on 11 GM species in the reverse MR analysis. Notably, Phylum *Proteobacteria* and DN are mutually causalities.

**Conclusion:**

This study identified the causal association between GM and DN with MR analysis, which may enhance the understanding of the intestinal-renal axis and provide novel potential targets for early non-invasive diagnosis and treatment of DN.

## 1 Introduction

Diabetes Nephropathy (DN) is widely recognized as the leading factor of Chronic Kidney Disease (CKD) and is considered one of the microvascular complications of Diabetes Mellitus (DM) (Gnudi et al., 2016). Around 30 to 40% of individuals with diabetes eventually develop DN. The prevalence of DN has demonstrated a consistent upward trend, paralleling the evolution of society and economy, alongside shifts in individual lifestyles and dietary patterns (Saeedi et al., 2019). DN is one of the key factors of end-stage renal disease (ESRD), contributing to around 54% of newly diagnosed incidents of ESRD and 30% of individuals requiring maintenance dialysis (Zhang L. et al., 2020). Moreover, DN can result in severe cardiovascular complications (Verma et al., 2020). The pathogenesis of DN is characterized by complex interplays of several factors (Samsu, 2021). At present, the treatment of DN mainly involves glycemic control and the use of pharmacological interventions, including angiotensin II receptor blockers (ARBs) or angiotensin-converting enzyme inhibitors (ACEIs), aimed at the regulation of the conditions (Doshi and Friedman, 2017; Cole and Florez, 2020). However, the risk of developing ESRD remains quite high (Anders et al., 2018; de Boer et al., 2020; Samsu, 2021). Therefore, enhancing our understanding of the pathogenic mechanisms of DN and exploring alternative therapeutic targets is imperative.

The human intestinal microbiome is described as the “second genome” that regulates health (Gilbert et al., 2018). Gut microbiota (GM) has been found to participate in the pathogenesis of various ailments by influencing the permeability of the intestinal barrier, the inflammatory response, and the balance of the immunological microenvironment (Lehto and Groop, 2018). Increasing evidence has suggested that the gut microbiota and kidney diseases can reciprocally influence each other through the induction of metabolic, immunological, and endocrine changes, a phenomenon commonly referred to as the “intestinal-renal axis” (Altamura et al., 2023). Several studies have demonstrated the substantial importance of gut microbial dysbiosis in the initiation and deterioration of DN (Wang Y. et al., 2022; Zhang et al., 2022; Zhao et al., 2023). It has been noted in several studies of GM particular alterations in the intestinal microbial composition of patients with DN, including the increase in the abundance of *Clostridium* and *Aspergillus*, and the decrease in the level of *Rhodococcus* (Salguero et al., 2019; Du et al., 2021; Wang Y. et al., 2022). Additionally, studies have demonstrated that a multitude of factors can contribute to the impairment of intestinal mucosa and an increase in permeability among individuals with DN, which allows the entry of metabolites such as indole and p-cresol into the bloodstream, subsequently instigating renal injury (Lehto and Groop, 2018; Lau et al., 2021). Numerous animal experiments have shown that pharmaceutical interventions induced alterations in the gut microbial composition of DN mice, subsequently leading to an amelioration of the disease’s attributes. This enhancement could potentially be attributed to mechanisms including the reduction of lipopolysaccharide (LPS)-producing microbes and the augmentation of short-chain fatty acids (SCFAs)-producing microbes (Chen et al., 2022; Deng et al., 2022).

The correlation between DN and GM has been well-established, and substantial research has been undertaken to unravel the potential mechanisms and corresponding therapeutic strategies involving GM and the kidney injury induced by their metabolites. However, the majority of these studies have mainly relied on animal models and cross-sectional investigations. Furthermore, the human GM is a complex and extensive ecosystem, which creates difficulties in identifying the causal association between certain GM and DN.

The Mendelian randomization (MR) analysis method has been widely applied in the field of epidemiological causal inference in recent years (Lehto and Groop, 2018). This method of analysis, based on Mendel’s second law, employed single-nucleotide polymorphisms (SNPs) associated with clinical phenotypes as Instrumental Variables (IVs) to build models so as to identify the causal association between exposures and outcomes at the genomic level (Davey Smith and Hemani, 2014). It achieves this without factoring in the influence of confounding variables (Holmes et al., 2017). The reliability of MR analysis has been substantiated by multiple studies. Furthermore, several studies have successfully utilized MR analysis to identify causal relationships between various exposures and outcomes successfully (Yeung and Schooling, 2021; Liu et al., 2022; Ma et al., 2022).

In this study, we aim to assess the causal association between GM and DN by employing MR analysis, thereby enhancing our understanding of the intestinal-renal axis and unveiling novel therapeutic targets for the early non-invasive diagnosis and treatment of DN.

## 2 Materials and methods

### 2.1 Study design

**FIGURE 1:**
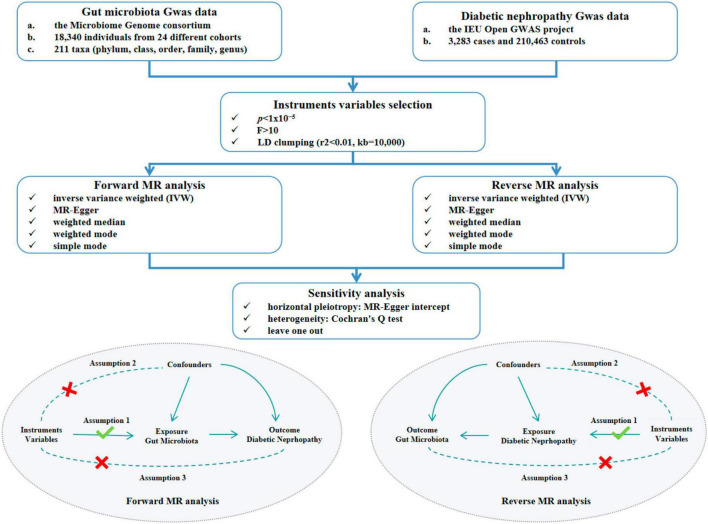
Study design and flow chart of MR analysis. MR, mendelian randomization. GWAS, genome-wide association study. LD, linkage disequilibrium.

This study explored the causal association between GM and DN, concurrently validating the robustness of the results through two-sample MR analysis (Figure 1). Three crucial assumptions of MR were satisfied to ensure the utmost accuracy throughout the entire process. Firstly, the selected IVs should demonstrate significant associations with the outcome. Secondly, IVs should demonstrate independence from any conceivable confounding factors that may affect both exposure and outcome. Thirdly, IVs should only influence the outcome via the exposure.

### 2.2 Data source

The genome-wide association studies (GWAS) summary data for human GM were acquired from the Microbiome Genome (MiBioGen) consortium. These data were obtained from an extensive multi-ethnic GWAS meta-analysis including a total of 18,340 individuals of European descent from 24 different cohorts. The investigation specifically focused on the GM, with a total of 211 distinct microbial species documented (Kurilshikov et al., 2021). The GWAS data could be accessed via the following URL.^1^ A total of 211 intestinal bacterial species were chosen as exposure factors and subsequently classified into five distinct biological categories, including phylum, class, order, family, and genus. DN was regarded as the outcome in this study. The statistics were acquired from the IEU Open GWAS project database, which contained a sample size of 3,283 cases and 210,463 controls originating from European populations, with a total of 16,380,453 SNPs identified. Diabetic nephropathy was identified as an outcome when glomerular disorders in individuals with diabetes mellitus, in accordance with the ICD-10 criteria (code: N08.3*). The GWAS data for DN could be accessible through the following link.^2^

### 2.3 Selection of instruments variables

For the purpose of obtaining qualified IVs, a further screening was conducted for the SNPs identified by GWAS. The SNPs used for MR analysis had to be strongly linked with the exposure, satisfying the association assumption of MR. Firstly, to ensure the inclusion of a satisfactory number of IVs, we selected SNPs with *p*-value below the locus-wide significance level (1x10^–5^). In addition, the formula F=(R^2^/(R^2^−1))×((N−K−1)/K) was utilized to compute the F value and IVs exhibiting F value below 10 were subsequently omitted, thereby addressing potential bias associated with weak IVs and ensuring the robustness of the association between the selected IVs and exposure factors (Li et al., 2023). This approach aims to prevent deviations and to enhance the validity of the findings. Thirdly, to ensure the independence of the IVs from one another, the clumping process was executed utilizing the TwoSampleMR package within the R software, in accordance with the criterion of Linkage Disequilibrium (LD). This step employed parameter settings with a SNP linkage disequilibrium value (*r*^2^) threshold of 0.01 and a clumping distance value of 10,000kb.

### 2.4 Mendelian randomization analysis

The inverse variance weighted (IVW) method was the main method employed in this study, in order to detect the potential causal association between GM and DN. In the meanwhile, four verified methods—MR-Egger, weighted median, weighted mode, and simple mode—were employed to give a thorough review of potential association in order to enhance the robustness of the results. In cases of inconsistent results, we prioritized IVW as the primary result. The IVW method utilizes a ratio approach to infer the causal impact of exposure on the outcome by weighted linear regression models under the assumption that the intercept term of IVs was zero. Notably, the IVW method has better efficacy and precision, when there is no horizontal pleiotropy among IVs, resulting in unbiased estimates of the status (Davey Smith and Hemani, 2014). The results were considered to be significant if the p-value of IVW was below 0.05, suggesting a causal association between the exposure and the outcome. The MR-Egger method employs weighted regressions that consider the inclusion of an intercept term, in contrast to the IVW method. The intercept term is utilized to assess the extent of multicollinearity among IVs, and the slope is the estimated value of the causal effect. The weighted median method reduces the rate of type I error and also accommodates the potential failures of certain genetic variants. The validity of the weighted mode method remains unaffected, in cases where the majority of IVs with comparable causal effect are valid, even if some IVs do not meet the criteria set by the MR method for causal inference (Xiang et al., 2021). Lastly, the simple mode method is less potent than the IVW method, but it still contributes to the robustness of our findings (Holmes et al., 2017).

### 2.5 Sensitivity analysis

The results of MR analysis may produce erroneous results influenced by weak IVs, genetic pleiotropy and other underlying issues. Therefore, we conducted sensitivity analysis to ascertain the stability of the outcomes. In this study, The MR-Egger method was employed to ascertain the existence of horizontal pleiotropy. The MR-Egger method has become a commonly adopted approach to examine the presence of horizontal pleiotropy by utilizing the intercept term of MR-Egger regression (Wang F. et al., 2023). If the intercept’s *p*-value exceeds 0.05, the horizontal pleiotropy is not statistically significant, and the exclusionary hypothesis holds true (Yavorska and Burgess, 2017; Yeung and Schooling, 2021). The Cochran’s Q test was conducted to detect the presence of heterogeneity among IVs for both the IVW and MR-Egger methods (Bowden et al., 2017; Liu et al., 2022; Ma et al., 2022). And if *p*-value exceeds 0.05, the influence of heterogeneity on the causal effect could be disregarded. Conversely, when heterogeneity is statistically significant (*p* < 0.05), the IVW random-effects estimator is utilized to mitigate the impact of heterogeneity on causal effects (Ren et al., 2023). In addition, in this study, the leave-one-out method was utilized to do the sensitivity analysis of the results to assess whether the causal effects observed in the MR analysis were caused by any single IV (Ren et al., 2023). The procedure entailed removing each SNP individually and then comparing the results obtained before and after the removal to determine whether there was a statistical significance. If *p*-value exceeded 0.05 derived after excluding an SNP suggested that the SNP did not have a non-specific influence on the effect estimates (Hemani et al., 2018). The “TwoSampleMR” R package (version 0.5.6, Stephen Burgess, Chicago, IL, USA) was utilized to perform two-sample MR analysis between exposure and outcome.

### 2.6 Reverse mendelian randomization analysis

A reverse MR analysis was conducted to detect the causal effect of DN on GM utilizing five MR methods. And the robustness of the results was validated through sensitivity analysis.

## 3 Results

### 3.1 The selection of instrumental variables

A comprehensive screening process was undertaken on a total of 211 distinct IVs representing diverse taxa within GM. Following meticulous scrutiny, IVs exhibiting cascading disequilibrium effects and those demonstrating a susceptibility to weak instrumental variable bias were excluded. Consequently, a total of 2,280 IVs emerged, meeting the threshold for significant locus-wide association (*p* < 10^–5^). These IVs were derived from diverse taxonomic classes within the GM, encompassing 9 phyla, 20 orders, 34 families, 16 classes, and 128 genera. Elaborated information can be referenced in Supplementary Table 1.

### 3.2 Mendelian randomization analysis

**FIGURE 2:**
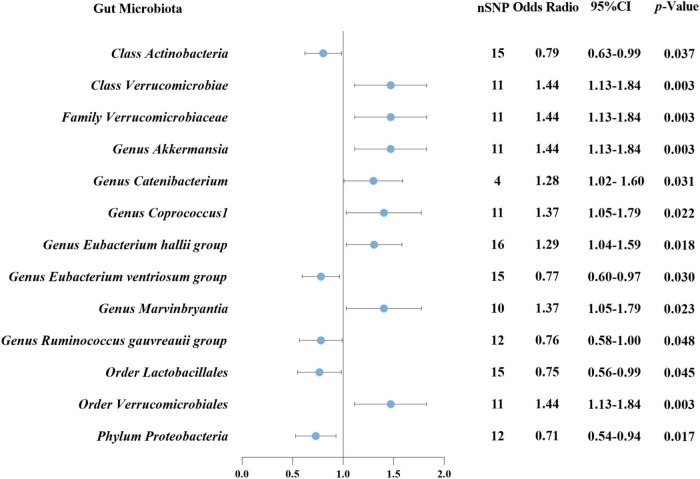
Causal association between GM and DN using inverse variance weighted method. GM, gut microbiota; DN, diabetic nephropathy; nSNP, number of single-nucleotide polymorphism; CI, confidence interval.

**FIGURE 3:**
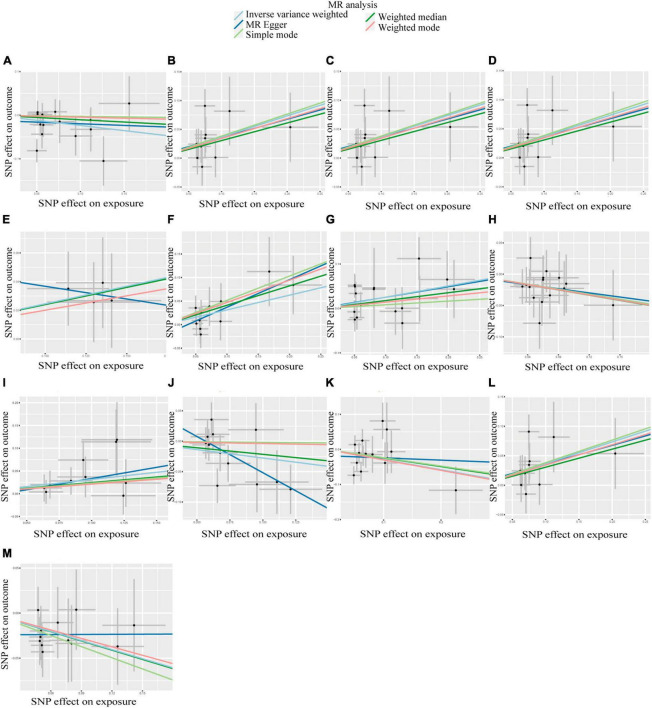
Scatter plots of the causal association between GM and DN. **(A)** Class *Actinobacteria*; **(B)** Class *Verrucomicrobiae*; **(C)** Family *Verrucomicrobiaceae*; **(D)** Genus *Akkermansia*; **(E)** Genus *Catenibacterium*; **(F)** Genus *Coprococcus* 1; **(G)** Genus *Eubacterium hallii* group; **(H)** Genus *Eubacterium ventriosum* group; **(I)** Genus *Marvinbryantia*; **(J)** Genus *Ruminococcus gauvreauii* group; **(K)** Order *Lactobacillales*; **(L)** Order *Verrucomicrobiales*; **(M)** Phylum *Proteobacteria*.

**FIGURE 4:**
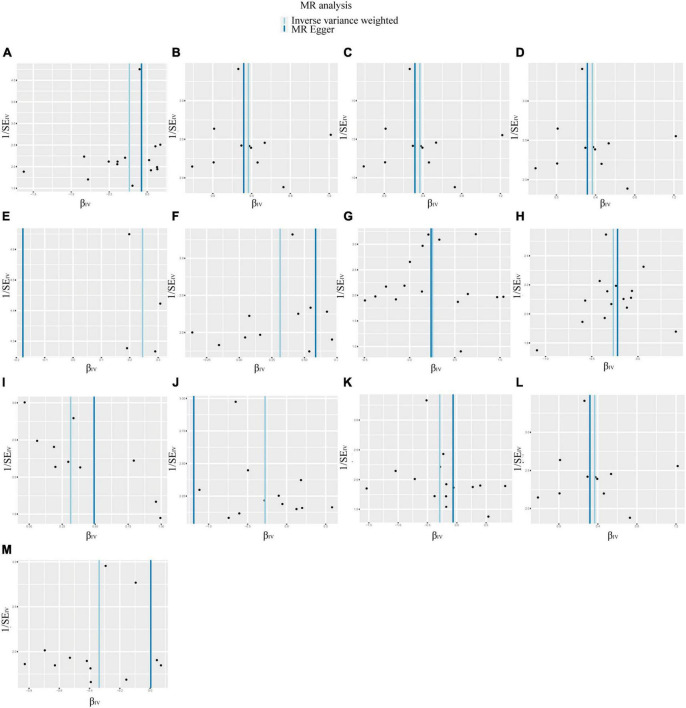
Funnel plots of the causal association between GM and DN. No significant bias in the results was demonstrated. **(A)** Class *Actinobacteria*; **(B)** Class *Verrucomicrobiae*; **(C)** Family *Verrucomicrobiaceae*; **(D)** Genus *Akkermansia*; **(E)** Genus *Catenibacterium*; **(F)** Genus *Coprococcus* 1; **(G)** Genus *Eubacterium hallii* group; **(H)** Genus *Eubacterium ventriosum* group; **(I)** Genus *Marvinbryantia*; **(J)** Genus *Ruminococcus gauvreauii* group; **(K)** Order *Lactobacillales*; **(L)** Order *Verrucomicrobiales*; **(M)** Phylum *Proteobacteria*.

According to IVW analysis, Class *Verrucomicrobiae* (OR: 1.44, 95%CI: 1.13–1.84, *p* = 0.003), Order *Verrucomicrobiales* (OR: 1.44, 95%CI: 1.13–1.84, *p* = 0.003), Family *Verrucomicrobiaceae* (OR: 1.44, 95%CI: 1.13–1.84, *p* = 0.003), Genus *Akkermansia* (OR: 1.44, 95%CI: 1.13–1.84, *p* = 0.003), Genus *Catenibacterium* (OR: 1.28, 95%CI: 1.02–1.60, *p* = 0.031), Genus *Coprococcus* 1 (OR: 1.37, 95%CI: 1.05–1.79, *p* = 0.022), Genus *Eubacterium hallii* group (OR: 1.29, 95%CI: 1.04–1.59, *p* = 0.018), and Genus Marvinbryantia (OR: 1.37, 95%CI: 1.05–1.79, *p* = 0.023) were associated with a higher risk of DN. On the contrary, Class *Actinobacteria* (OR: 0.79, 95%CI: 0.63–0.99, *p* = 0.037), Genus *Eubacterium ventriosum* group (OR: 0.77, 95%CI: 0.60–0.97, *p* = 0.030), Genus *Ruminococcus gauvreauii* group (OR: 0.76, 95%CI: 0.58–1.00, *p* = 0.048), Order *Lactobacillales* (OR: 0.75, 95%CI: 0.56–0.99, *p* = 0.045), Phylum *Proteobacteria* (OR: 0.71, 95%CI: 0.54–0.94, *p* = 0.017) were associated with a lower risk of DN, as shown in Figure 2. The weighted median analysis showed that the Genus *Coprococcus* 1 (OR: 1.51, 95%CI: 1.06–2.16, *p* = 0.024) was causally associated with a higher risk of DN. MR-Egger analysis showed that the Genus *Ruminococcus gauvreauii* group was causally associated with a lower risk of DN. However, no significant associations were discerned in the outcomes of the remaining analyses. A comparative overview of the results from the other four MR analyses is delineated in Supplementary Table 2, and the scatter plots of the five MR analyses are depicted in Figure 3. The funnel plots of the IVW and MR-Egger analysis are depicted in Figure 4, and no significant bias in the results was demonstrated.

### 3.3 Sensitivity analysis

**FIGURE 5:**
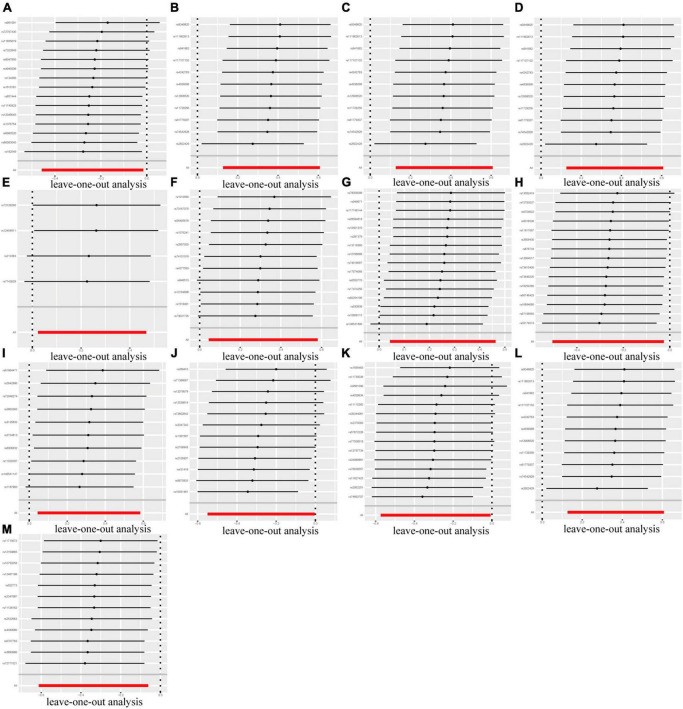
Leave-one-out plots of the causal association between GM and DN. The established causal association was unlikely to be influenced by any specific SNP. **(A)** Class *Actinobacteria*; **(B)** Class *Verrucomicrobiae*; **(C)** Family *Verrucomicrobiaceae*; **(D)** Genus *Akkermansia*; **(E)** Genus *Catenibacterium*; **(F)** Genus *Coprococcus* 1; **(G)** Genus *Eubacterium hallii* group; **(H)** Genus *Eubacterium ventriosum* group; **(I)** Genus *Marvinbryantia*; **(J)** Genus *Ruminococcus gauvreauii* group; **(K)** Order *Lactobacillales*; **(L)** Order *Verrucomicrobiales*; **(M)** Phylum *Proteobacteria*.

In the context of sensitivity analysis, the MR-Egger regression analysis indicated that the *p*-value of the intercept associated with the selected IVs lacked statistical significance (*p* > 0.05), suggesting that the chosen IVs were not subject to horizontal pleiotropy (Table 1). Furthermore, the heterogeneity of the IVs was assessed utilizing Cochran’s Q test, and the results of neither the IVW nor the MR-Egger analyses yielded statistically significant results (*p* > 0.05), indicating an absence of significant heterogeneity among the selected IVs (Table 1). Lastly, the outcomes of the leave-one-out method are depicted in Figure 5. The outcomes implied that the established causal association was unlikely to be influenced by any specific SNP.

**TABLE 1:** The results of tests for horizontal pleiotropy and heterogeneity in toward MR analysis. No horizontal pleiotropy or heterogeneity was found among instruments variables. MR, mendelian randomization. IVW, inverse variance weighted.

Gut microbiota	MR-Egger regression		Heterogeneity (IVW)		Heterogeneity (MR-Egger)	
	Egger intercept	*p*-Value	Cochran’s Q	*p*-Value	Cochran’s Q	*p*-Value
**Class**						
*Actinobacteria*	−0.012	0.615	13.806	0.464	13.530	0.408
*Verrucomiiae*	0.004	0.904	8.609	0.570	8.593	0.476
**Family**						
*Verrucomicrobiaceae*	0.004	0.901	8.616	0.569	8.600	0.475
**Genus**						
*Akkermansia*	0.004	0.900	8.618	0.569	8.601	0.475
*Catenibacterium*	0.054	0.794	0.215	0.975	0.126	0.939
*Coprococcus* 1	−0.022	0.399	5.081	0.886	4.298	0.891
*Eubacterium hallii* group	0.001	0.937	14.293	0.503	14.286	0.429
*Eubacterium ventriosum*	−0.003	0.933	5.826	0.971	5.818	0.953
*Marvinbryantia*	−0.016	0.743	4.939	0.840	4.823	0.776
*Ruminococcus gauvreauii*	0.067	0.106	13.543	0.259	10.287	0.416
**Order**						
*Lactobacillales*	−0.018	0.529	18.215	0.197	17.647	0.171
*Verrucomicrobiales*	0.004	0.904	8.609	0.570	8.593	0.476
**Phylum**						
*Proteobacteria*	−0.024	0.373	3.484	0.983	2.615	0.989

### 3.4 Reverse mendelian randomization analysis

**FIGURE 6:**
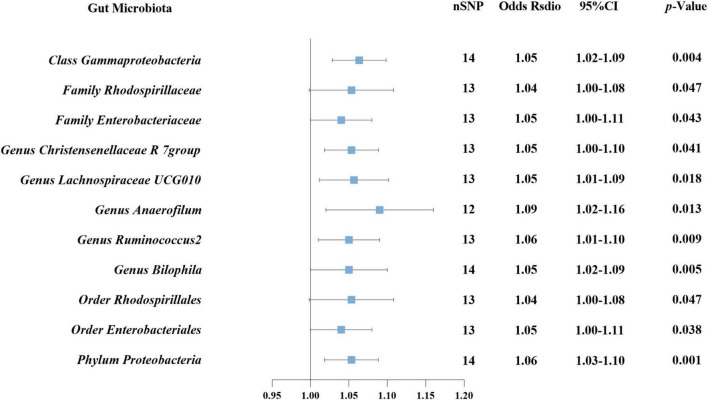
Causal association between DN and GM using inverse variance weighted method.

In reverse MR analysis, causal effects were found of DN on 11 GM species utilizing IVW method (Figure 6), including Class *Gammaproteobacteria*, Family *Rhodospirillaceae*, Family *Enterobacteriaceae*, Genus *Christensenellaceae* R 7group, Genus *Lachnospiraceae* UCG010, Genus *Anaerofilum*, Genus *Ruminococcus* 2, Genus *Bilophila*, Order *Rhodospirillales*, Order *Enterobacteriales*, and Phylum *Proteobacteria*. Notably, Phylum *Proteobacteria* and DN were mutually causalities. The results of the other four MR analysis are delineated in Supplementary Table 3, and the scatter plots of the five MR analyses are depicted in Supplementary Figure 1. The funnel plots of the IVW and MR-Egger analyses are depicted in Supplementary Figure 2, and no significant bias of the results is demonstrated. The sensitivity analysis indicated no significant horizontal pleiotropy and heterogeneity among the selected IVs (Table 2). Lastly, the outcomes of the leave-one-out method implied that the established causal association was unlikely to be influenced by any specific SNP (Supplementary Figure 3).

**TABLE 2:** The results of tests for horizontal pleiotropy and heterogeneity in reverse MR analysis. No horizontal pleiotropy or heterogeneity was found among instruments variables.

Gut microbiota	MR-Egger regression		Heterogeneity (IVW)		Heterogeneity (MR-Egger)	
	Egger intercept	*p*-Value	Cochran’s Q	*p*-Value	Cochran’s Q	*p*-Value
**Class**						
*Gammaproteobacteria*	−0.001	0.937	5.635	0.958	5.628	0.934
**Family**						
*Rhodospirillaceae*	−0.006	0.649	10.495	0.573	10.277	0.506
*Enterobacteriaceae*	0.003	0.773	6.987	0.858	6.900	0.807
**Genus**						
*Christensenellaceae* R 7group	0.003	0.720	12.589	0.480	12.449	0.410
*Lachnospiraceae* UCG010	−0.011	0.274	10.664	0.558	9.336	0.591
*Anaerofilum*	−0.006	0.685	8.597	0.659	8.423	0.588
*Ruminococcus* 2	−0.015	0.116	13.948	0.304	11.027	0.441
*Bilophila*	−0.004	0.718	15.278	0.227	15.089	0.178
**Order**						
*Rhodospirillales*	−0.008	0.536	0.499	0.499	10.943	0.448
*Enterobacteriales*	0.003	0.773	6.987	0.858	6.900	0.807
**Phylum**						
*Proteobacteria*	0.002	0.794	10.963	0.614	10.891	0.538

## 4 Discussion

There has been a rise in the prevalence of both diabetes and DN in recent years. According to the International Diabetes Federation, there will be the largest DM population in China, India, and the United States by the year of 2030, the number of which will hit 140 million, 101 million, and 34 million respectively (Saeedi et al., 2019). Concurrently, along with the proposition of concept of the gut-kidney axis and the subsequent in-depth investigations therein, the substantive role of GM dysbiosis in the intricate pathogenesis of DN has been progressively unveiled. Despite these progressions, the conclusive establishment of a causal association between GM and DN remains an unresolved inquiry. In this study, MR analysis was employed to comprehensively identify the underlying causal association between GM and DN. The results of this study elucidated causal associations between thirteen distinct GM species and DN. Class *Verrucomicrobiae*, Order *Verrucomicrobiales*, Family *Verrucomicrobiaceae*, Genus *Akkermansia*, Genus *Catenibacterium*, Genus *Coprococcus* 1, Genus *Eubacterium hallii* group and Genus *Marvinbryantia* were associated with an elevated risk of DN. Conversely, Class Actinobacteria, Group *Eubacterium ventriosum* group, Group *Ruminococcus gauvreauii* group, Order *Lactobacillales*, Phylum *Proteobacteria* were associated with a reduced risk of DN. Notably, by integrating the results of this study with previous research, we found that that dysbiosis of the gut microbiota and its metabolic byproducts play significant roles in the progression of DN through various mechanisms.

Among patients with DN, the co-occurrence of systemic inflammation and compromised innate immunity has been noted (Han et al., 2023). Research has indicated that the gut microbiota, inhabiting the gastrointestinal tract, can modulate antigen reactivity in lymphoid tissues, thus initiating and gradually maturing the intestinal immune system (Cerf-Bensussan and Eberl, 2012). Specifically, genera such as *Bacteroides*, *Bifidobacterium*, *Lactobacillus*, and *Bacillus* proteus have been found to notably contribute to immune system enhancement (Wu and Wu, 2012). The dysregulation of the gut microbiota can influence the maturation of macrophages, prompting the release of tumour necrosis factor-alpha (TNF-α) and interleukin-6 (IL-6) upon Toll-like receptor (TLR) stimulation, thereby triggering renal inflammation (Yang et al., 2019). Additionally, this imbalance may activate innate immune cells, amplifying the activity of TLR-2 and TLR-4 pathways, and fostering the production of inflammatory cytokines. Such dysbiosis in patients with DN could potentially lead to immune dysfunction and renal injury (Lin et al., 2012; Mudaliar et al., 2013; Donaldson et al., 2016; Chi et al., 2021). Concurrently, compromised immune function may diminish the body’s defence capabilities and heighten susceptibility to infections (Robinson and Freedman, 2018). Urinary tract infections, a frequent complication in individuals with diabetes, may be linked to systemic inflammation associated with hyperglycaemia and dysbiosis of the gut microbiota, thereby exacerbating renal damage in DN (Micle, 2020; Wang C. et al., 2023).

The GM exerted an impact on the body’s metabolic processes and obesity via insulin resistance (IR) (Hoseini Tavassol et al., 2023). Previous research underscored the positive correlation between the severity of IR and the incidence of DN (Penno et al., 2021). Furthermore, it was established that IR contributed to the pathogenesis of DN independently of hyperglycaemia, as it could elicit an increased salt sensitivity (Karalliedde and Gnudi, 2016). Simultaneously, IR led to a reduction in glucose transport by podocytes, which could precipitate the disruption of the glomerular filtration barrier and the onset of proteinuria (Alqallaf et al., 2022).

SCFAs represent a subset of saturated fatty acids characterized by no more than six carbon atoms, predominantly encompassing acetic, propionic, and butyric acids (Zhang et al., 2021). And SCFAs are generated by GM. These chemicals have multifaceted functions, primarily in the regulation of energy metabolism, maintenance of the intestinal epithelial barrier, facilitation of immune responses, and modulation of inflammatory processes (Du et al., 2022). Prior investigations demonstrated diminished serum concentrations of total SCFAs in individuals diagnosed with DN (Zhong et al., 2021; Cai et al., 2022). SCFAs exerted their influence as signalling moieties, engaging a dedicated G-protein-coupled receptor 43 (GPR43), in which activation of GPR43 signalling instigated a regulation of inflammatory cascades. This effect manifested as a reduction in levels of pro-inflammatory cytokines within the tissues of the colon, thus contributing to the preservation of intestinal homeostasis (Sun et al., 2017; Priyadarshini et al., 2018). Moreover, SCFAs can augment neutrophil chemotaxis and facilitate the differentiation and proliferation of natural killer cells and regulatory T cells, thus activating the immune system (Kim et al., 2014). Concurrently, the augmentation in the abundance of urease-producing bacteria precipitated a surge in intestinal pH, consequently elevating the permeability of the intestinal mucosa (Lehto and Groop, 2018; Thaiss et al., 2018; Lau et al., 2021). This facilitated the entry of specific uremic toxin precursors, including cresol, indole, and trimethylamine into the systemic circulation, thereby facilitating the production of uremic toxins. These entities, in turn, elicited oxidative stress and fostered renal tubulointerstitial fibrosis, resulting in a progressive decline in renal function (Kikuchi et al., 2019; Tan et al., 2021). However, SCFAs can preserve intestinal homeostasis and consequently mitigate the absorption of harmful substances through maintaining the structural integrity of the intestinal mucosa, thus bestowing a protective impact on renal function. Furthermore, it has been revealed that SCFAs, especially butyric acid, can enhance tight junction protein complex, thereby maintaining the perpetuation of the intestinal barrier’s functional integrity (Xia et al., 2020). This mechanistic facet holds potential in averting renal impairments. Furthermore, a hyperglycaemic state typically caused the body to generate an overproduction of reactive oxygen species (ROS), resulting in the development of DN (Miranda-Díaz et al., 2016). It was proposed that SCFAs had the capacity to inhibit the stimulation of glomerular mesangial cells caused by hyperglycaemia and lipopolysaccharides (LPS). Concurrently, SCFAs were found to diminish the generation of ROS and malondialdehyde (MDA), along with inflammatory factors, while elevating the level of superoxide dismutase (SOD). These activities mitigated inflammatory responses, ultimately safeguarding renal function (Zhang et al., 2022). Previous research outlined the role of SCFAs in mitochondrial biosynthesis processes (Andrade-Oliveira et al., 2015). This finding suggested that SCFAs could potentially have a positive impact on alleviating renal epithelial cell hypoxia. Moreover, SCFAs treatment improved renal insufficiency in a murine model of acute kidney injury subsequent to renal ischemia-reperfusion (Antza et al., 2018).

Prior research identified an increased prevalence of the Order Lactobacillales and the Class Actinobacteria in the intestines of patients with DN (Du et al., 2021; Wang Y. et al., 2022). This observation was attributed to the use of hypoglycaemic medications (Forslund et al., 2015; Gu et al., 2017). Bifidobacterium and Lactobacillus are widely acknowledged as significant intestinal probiotics (Bindels et al., 2015; Markowiak-Kopeć and Śliżewska, 2020), generating lactic acid and acetic acid within the intestinal environment, thereby safeguarding the host from invasion by intestinal pathogens (Zhang et al., 2015; Roy and Dhaneshwar, 2023). It was found that an elevation in the abundance of the Class Actinobacteria could lead to heightened production of SCFAs, primarily due to the presence of Bifidobacterium (Binda et al., 2018). Lactobacillus, a notable constituent of the Order Lactobacillales, possesses the capability to convert lactic acid into diverse SCFAs forms. In patients with CKD, a reverse relationship was observed between Lactobacillus levels and markers of kidney impairment, including blood creatinine and urea nitrogen (Ren et al., 2020). These findings imply that Order Lactobacillales and Class Actinobacteria serve as protective factors against DN. This concurs with our own research findings and deepens the understanding of the pivotal role these microbiotas play in DN.

The Phylum Proteobacteria represents one of the most expansive and phenotypically diverse phyla within the field of bacteria (Sharma et al., 2022), and it is considered to have the potential for pathogenicity under specific circumstances (Du et al., 2021). The findings from the present investigation elucidate a correlation between this particular bacterial cohort and a lower risk of DN. Previous research demonstrated an association between the Phylum Proteobacteria and an increased synthesis of SCFA production (Machate et al., 2020). Moreover, an alteration in the abundance of the Phylum Proteobacteria was observed within the intestinal environment of patients with DN, exhibiting a disparity when compared to the composition found in healthy populations (Wang Y. et al., 2022). In alignment with the outcomes of the current study, these findings propose that this genus is linked with and held the potential to function as a protective factor for the progression of DN.

The Genus Eubacterium ventriosum group is a component of the Family *Lachnospiraceae*. The Genus *Ruminococcus gauvreauii* group belongs to the Family *Ruminococcaceae* and is a component of the Phylum *Firmicutes* (Zhang et al., 2023). The association between these two genera and DN has not been examined by conventional epidemiological studies. Nevertheless, the outcomes derived from the present study suggest that both genera are associated with a reduced risk of DN. This assertion is likely attributable to their membership within the community of microbiota known for producing SCFAs (Kemp et al., 2021; Amamoto et al., 2022; Wang J. et al., 2023). This discovery offers a novel perspective on the involvement of these two genera in DN.

In healthy individuals, the Genus Akkermansia (Class *Verrucomicrobiae*, Order *Verrucomicrobiales*, Family *Verrucomicrobiaceae*) typically constitutes approximately 3 and 5 % of the gastrointestinal microbial community (Hasani et al., 2021). Currently, the Genus *Akkermansia* represents the only Phylum *Verrucomicrobia* found in the human intestine. As a result, many 16S rRNA gene sequence analyses considered the Phylum *Verrucomicrobia* to be a representative of Genus *Akkermansia* (Cani et al., 2022). It has been demonstrated that there is a correlation between a reduction in the abundance of the Genus *Akkermansia*, a promising probiotic, and the development of various illnesses, including type 2 diabetes and inflammatory bowel disease (Wang K. et al., 2022). In addition, the level of Genus *Akkermansia* was negatively correlated with the level of IR (Shin et al., 2014; Watanabe et al., 2023). Moreover, it should be noted that Genus *Akkermansia* also has the capacity to produce SCFAs (Luo et al., 2022), but its role remains somewhat controversial. In animal models of DN and patients with CKD, the level of Genus *Akkermansia* was positively correlated with markers of kidney injury, including blood creatinine and urea nitrogen (Forslund et al., 2015; Lan et al., 2023). Additionally, it was also found that the relative abundance of Genus *Akkermansia* was positively correlated with the severity of the disease in patients with Parkinson’s disease and multiple sclerosis (Jangi et al., 2016; Cekanaviciute et al., 2017; Zhang F. et al., 2020). Based on the results of the current study, there was a positive association between Genus Akkermansia and the risk of developing DN. This may be due to the fact that the relative abundance of Gram-negative bacteria is increased in the gastrointestinal tract of patients with DN, including Genus Akkermansia. Gram-negative bacteria contain LPS as components of the outer membrane of the cell wall. As the intestinal barrier was compromised, LPS entered the circulation and stimulates the body to produce excessive pro-inflammatory factors (e.g., IL-1β, IL-6, and TNFα, etc.), which further exacerbated the systemic inflammatory response in patients with DN (Salguero et al., 2019). Finally, it was demonstrated that LPS could bind to TLR, thereby activating the TLR-4 signaling pathway. This activation subsequently triggered downstream signaling pathways, resulting in tissue damage through mechanisms including oxidative stress and DNA damage (Hung et al., 2017).

To date, no study has reported alterations in the abundance of Genus Catenibacterium, Genus *Coprococcus* 1, Genus *Eubacterium hallii* group, and Genus *Marvinbryantia* in the intestinal tract of DN patients. The Genus *Catenibacterium* belongs to the Family *Erysipelotrichaceae* (Ricci et al., 2023). According to the results of this study, the Genus Catenibacterium is a risk factor for DN. This genus was found to be associated with several metabolic diseases (Burakova et al., 2022). Moreover, an increased prevalence of this genus was identified in faecal samples from individuals with ESRD, suggesting a potential correlation between the levels of this genus and the progression of nephropathy (Vaziri et al., 2013). There are very few studies on the Genus *Coprococcus* 1. However, the results of the current study suggest that this genus is positively associated with the risk of DN, warranting comprehensive elucidation of its underlying mechanisms. Genus Eubacterium hallii group is a member of the Family Lachnospiraceae within the Phylum Firmicutes, and as one of the butyrate-secreting genera, it has the potential to ameliorate DM and other diseases associated with IR (Zhang et al., 2015). Similarly, an elevated abundance of Genus Marvinbryantia was found to align with diminished IR levels, which could also produce butyrate (Chen et al., 2021). This indicates the potential benefits of both Genus Eubacterium hallii group and the Genus Marvinbryantia in ameliorating the condition of DN. Nevertheless, the present study reveals a positive correlation between these microbiotas and the risk of DN, potentially attributable to complex gene-gene and gene-environment interactions.

To the best of our knowledge, this is the first study to employ MR to assess the causal association between GM and DN. The implementation of this analytical methodology serves to reduce potential biases arising from reverse causation and residual confounding. This study provides confirmation of a causal association between GM and DN while delving into the plausible mechanistic pathways of intestinal dysbiosis in DN. These findings underscore the significance for nephrologists to exercise vigilant attention over DN patients exhibiting intestinal dysbiosis in their clinical practice. Furthermore, the identification of distinct GM associated with DN within this study provides novel biomarkers for the prevention, diagnosis and therapeutic intervention of DN, thereby enhancing our comprehension of the gut-renal axis.

However, this study has several limitations. Firstly, the predominant participants within the GWAS cohort were European ancestry, which might potentially affect the applicability of study findings across diverse ethnicities. Secondly, the available data pertaining to GM were solely categorized at a taxonomic level higher than the genus, hence limiting the ability to identify causal connections between GM and DN at more specific taxonomic levels such as species or strain. Additionally, there may be a partial overlap of data on SNPs present in GM across distinct taxonomic tiers, which may impact the reproducibility of the results of MR analyses. Lastly, some of the GM identified in this study have not been previously identified as directly associated with DN, and the findings of this study of certain GM fail to align with the results of prior studies. Hence, further population-based prospective studies and experiments are required to investigate the potential biological mechanisms between these GM and DN.

## 5 Conclusion

In conclusion, our study provides genetic insights into the potential causal relationships between specific GM and DN. We identified particular microbial communities with protective or detrimental roles in DN, thereby augmenting our understanding of the intricate interplay between the gut and kidneys in the development of DN. These findings offer valuable directions for future research and therapeutic interventions.

## Data availability statement

The datasets presented in this study can be found in online repositories. The names of the repository/repositories and accession number(s) can be found in the article/Supplementary material.

## Author contributions

YJ: Writing—review and editing, Writing—original draft. CH: Writing—review and editing. DY: Writing—review and editing, Visualization, Software, Methodology, Data curation. SG: Writing—review and editing, Supervision, Project administration.

## Footnotes

1
https://mibiogen.gcc.rug.nl/

2
https://gwas.mrcieu.ac.uk/datasets/finn-b-DM_NEPHROPATHY/
