# Supplementary material for: Association between gut microbiota and diabetic nephropathy: a mendelian randomization study
Source: Front Microbiol. 2024 Mar 27;15:1309871. doi: 10.3389/fmicb.2024.1309871 (PMC11004376; doi:10.3389/fmicb.2024.1309871)
Supplement: Supplementary file 4 [file Table_4.DOCX]

Supplementary Material

# Supplementary Data

Supplementary Material should be uploaded separately on submission. Please include any supplementary data, figures and/or tables.

Supplementary material is not typeset so please ensure that all information is clearly presented, the appropriate caption is included in the file and not in the manuscript, and that the style conforms to the rest of the article.

# Supplementary Figures and Tables

For more information on Supplementary Material and for details on the different file types accepted, please see [here](https://www.frontiersin.org/guidelines/author-guidelines#supplementary-material).

## Supplementary Figures


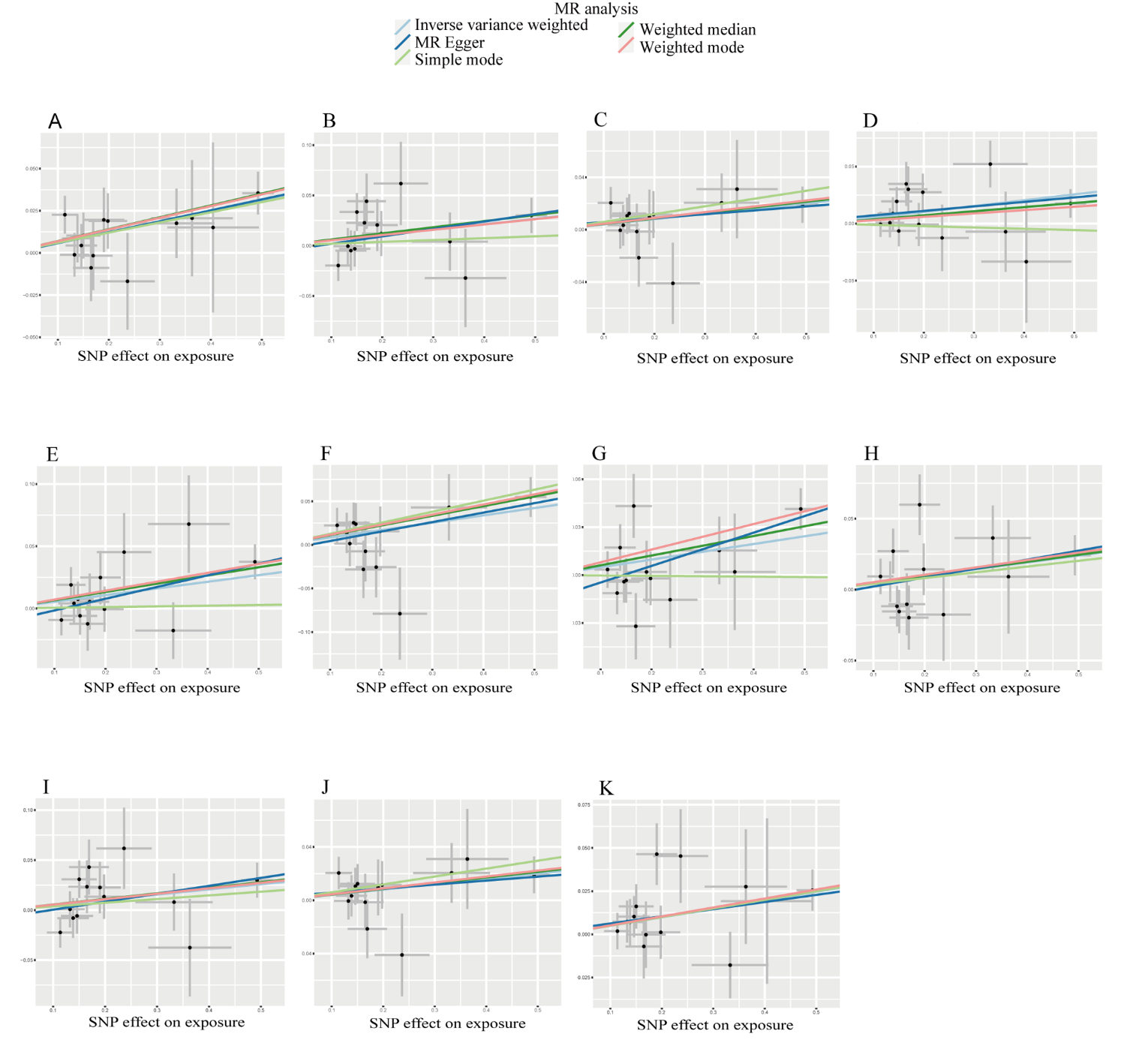


**Supplementary Figure 1.** Scatter plots of the causal association between diabetic nephropathy and gut microbiota. (**A**) Class Gammaproteobacteria; (**B**) Family Rhodospirillaceae; (**C**) Family Enterobacteriaceae; (**D**) Genus Christensenellaceae R 7group; (**E**) Genus Lachnospiraceae UCG010; (**F**) Genus Anaerofilum; (**G**) Genus Ruminococcus2; (**H**) Genus Bilophila; (**I**) Order Rhodospirillales; (**J**) Order Enterobacteriales; (**K**) Phylum Proteobacteria.


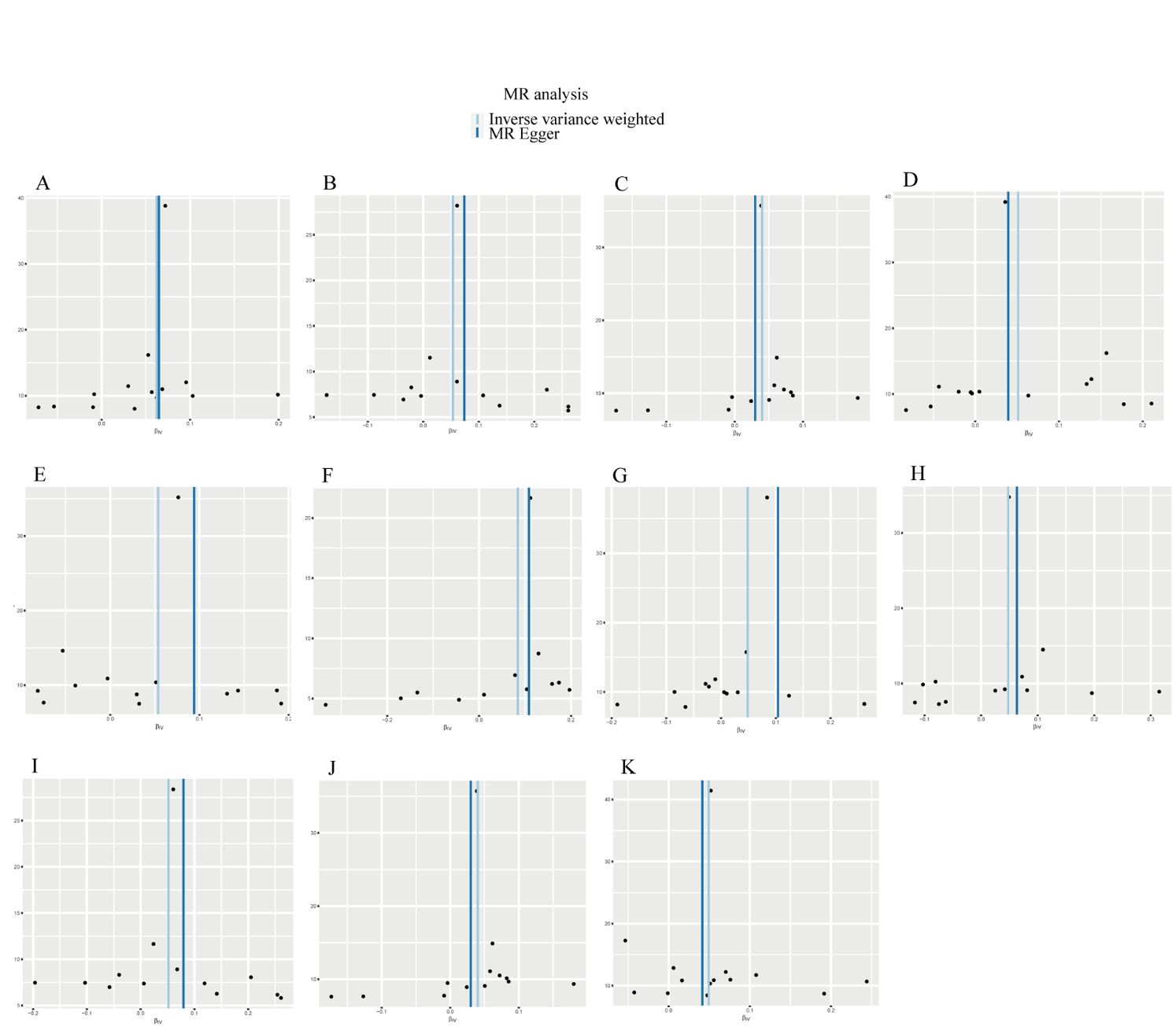


**Supplementary Figure 2.** Funnel plots of the causal association between diabetic nephropathy and gut microbiota. No significant bias in the results was demonstrated. (**A**) Class Gammaproteobacteria; (**B**) Family Rhodospirillaceae; (**C**) Family Enterobacteriaceae; (**D**) Genus Christensenellaceae R 7group; (**E**) Genus Lachnospiraceae UCG010; (**F**) Genus Anaerofilum; (**G**) Genus Ruminococcus2; (**H**) Genus Bilophila; (**I**) Order Rhodospirillales; (**J**) Order Enterobacteriales; (**K**) Phylum Proteobacteria.


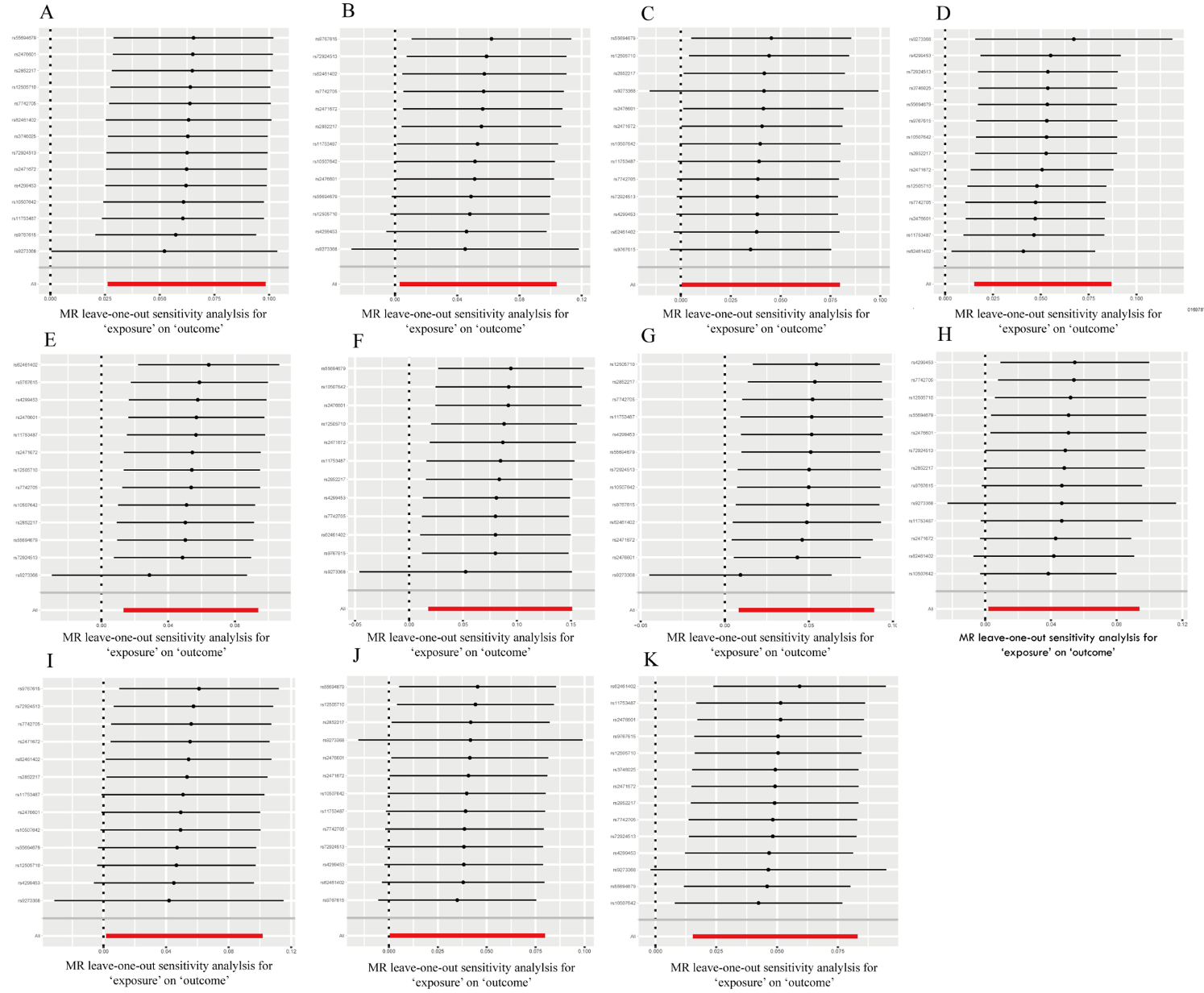


**Supplementary Figure 3.** Leave-one-out plots of the causal association between diabetic nephropathy and gut microbiota. The established causal association was unlikely to be influenced by any specific SNP. (**A**) Class Gammaproteobacteria; (**B**) Family Rhodospirillaceae; (**C**) Family Enterobacteriaceae; (**D**) Genus Christensenellaceae R 7group; (**E**) Genus Lachnospiraceae UCG010; (**F**) Genus Anaerofilum; (**G**) Genus Ruminococcus2; (**H**) Genus Bilophila; (**I**) Order Rhodospirillales; (**J**) Order Enterobacteriales; (**K**) Phylum Proteobacteria.
